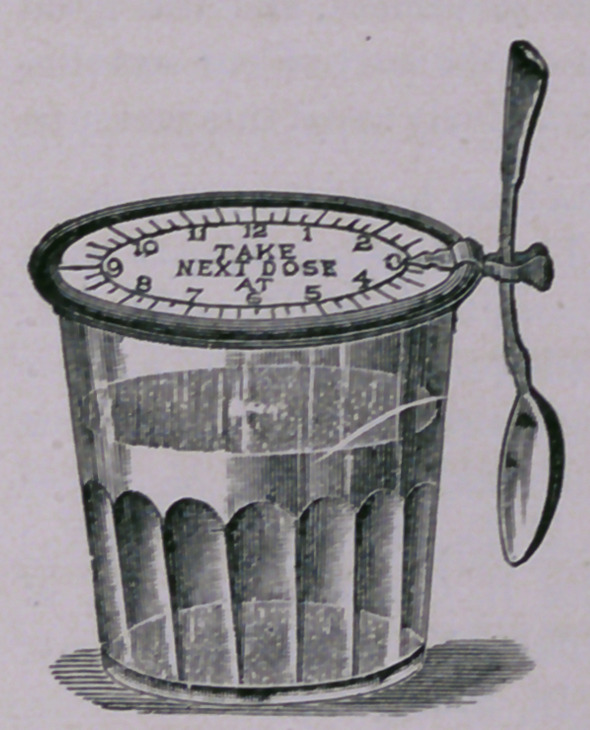# Notes and Notices

**Published:** 1896-07

**Authors:** 


### NOTES AND NOTICES.

A Medicine-Glass Cover.—This cut represents a new medicine-glass cover with index to show the hour when the next dose is to be taken and a spoonholder. It is particularly useful to homœopathic physicians and is highly indorsed by physicians of both schools. Price, 15 cents. Sharon Manufacturing Co., 17 North Juniper Street, Philadelphia, Pa.

Inter-State Committee of the American Institute.—At a session of the American Institute of Homœopathy, held in Newport, R. I., in June, 1895, the homœopathic society of each State was requested to appoint two of its members as delegates, to unitedly form an Inter-State Committee of this Institute. In the forty-five States of the Union, there already exist thirty-three such State Societies, twenty-eight of which appointed and reported such delegates. These delegates assembled at Detroit during the recent session of the Institute, organized and carefully considered the relations of the State Societies to the Institute and to each other.

In accordance with the recommendations of this Inter-State Committee, the Institute adopted the following preamble and recommendation:

WHEREAS, It is of great importance that our State Societies should be in harmony with the American Institute of Homœopathy, therefore, in order to secure this end,

WE RECOMMEND, The revival of the former custom by which the Presidents of our State Societies shall become honorary vice-presidents, and the secretaries, corresponding secretaries of the Institute, during their respective terms of office.

The following recommendations were also adopted:

1st. The legal incorporation of all homœopathic State Societies, not already incorporated;

2d. The organization and incorporation of homœopathic State Societies in States containing a sufficient number of homœopathic physicians, wherever no such organizations now exist;

3d. There is to be urged upon all homœopathic State Societies to annually furnish the Institute with correct lists of homœopathic physicians and of all homœopathic institutions (including hospitals, colleges, societies, journals, etc.), in their respective States; also, that an annual report of desirable locations for homœopathic physicians be prepared by the State Societies for publication, and that copies be furnished to the American Institute;

4th. That this Inter-State Committee be made a permanent Committee;

5th. That each State Society shall annually publish a list of its members, together with a *résumé* of its general transactions;

6th. That a system of the Inter-State delegations between our State Societies be arranged as far as practicable.

The earnest interest already exhibited in this movement, and the great importance of harmonious and systematic action on the part of our societies and institutions, should lead every State Society to actively assist this measure.

THE INTERNATIONAL HAHNEMANNIAN ASSOCIATION held its annual meeting at Glen Summit, Pa., June 24th and 25th. The following officers were elected for the ensuing year: President, Wm. P. Wesselhœft, M. D., of Boston; Vice-President, Walter M. James, M. D., of Philadelphia; Secretary, Erastus E. Case, M. D., of Hartford, Conn.; Corresponding Secretary, Mary Florence Taft, M. D., of Newtonville, Mass.; Treasurer, Franklin Powel, M. D., of Chester, Pa.; Board of Censors, B. L. B. Baylies, M. D., of Brooklyn, N. Y.; A. R. Morgan, M. D., of Waterbury, Conn.; Alice B. Campbell, M. D., of Brooklyn, N. Y.; E. P. Hussey, M. D., of Buffalo, N. Y.; L. A. L. Day, M. D., of Chicago; Necrologist, Stuart Close, M. D., Brooklyn, N. Y.

## Figures and Tables

**Figure f1:**